# Midterm clinical outcomes of mechanical versus rheolytic thrombectomy for iliofemoral or iliocaval deep vein thrombosis

**DOI:** 10.1016/j.jvsv.2026.102457

**Published:** 2026-02-05

**Authors:** Gabor Forgo, Silvia Cardi, Riccardo Fumagalli, Tim Sebastian, Daniel Périard, Stefano Barco, Nils Kucher, Rolf P. Engelberger

**Affiliations:** a Department of Angiology, University Hospital Zurich, Zurich, Switzerland; b Department of Biomedical Sciences, Humanitas University, Pieve Emanuele, Italy; c Unit of Angiology, HFR Fribourg, Hôpital Universitaire et Cantonal, Fribourg, Switzerland; d Center for Thrombosis and Hemostasis, University Medical Center Mainz, Mainz, Germany; e Faculty of Science and Medicine, University of Fribourg, Fribourg, Switzerland

**Keywords:** DVT, Mechanical thrombectomy, PTS, Rheolytic thrombectomy, Venous stent patency

## Abstract

**Objective:**

In patients with iliofemoral deep vein thrombosis (DVT), early thrombus removal reduces the risk of post-thrombotic syndrome (PTS). It remains uncertain if mechanical thrombectomy (MT) using the ClotTriever system may offer advantages as compared with rheolytic thrombectomy (RT) using the AngioJet ZelanteDVT.

**Methods:**

In our multicenter, retrospective, observational study, we included 122 patients (mean age, 48 years; 57% women) with iliofemoral (78%) or iliocaval DVT (22%). All underwent early thrombus removal with either MT (n = 40) or RT (n = 82) and had a minimum of 3 months of follow-up. Periprocedural outcomes included periprocedural thrombolytic use, access complications, and stent placement rate. Clinical outcomes included stent patency rate and freedom from PTS.

**Results:**

The median follow-up was 25 months (interquartile range, 11-52 months). Compared with RT, MT was associated with lower periprocedural thrombolytic use (38% vs 95%; *P* < .01) and a lower rate of stent placement (70% vs 98%; *P* < .01). Postprocedural access site thrombosis of the popliteal vein occurred in 5 MT patients (13%) and in none of the RT patients. At 1 year, primary and secondary patency rates were 80% (95% confidence interval [CI], 67%-95%) and 97% (95% CI, 93%-100%) in the MT group, and 88% (95% CI, 81%-96%) and 97% (95% CI, 94%-100%) in the RT group, respectively. Freedom from PTS at latest follow-up was observed in 98% of the MT group (95% CI, 93%-100%) and 94% of RT patients (95% CI, 87%-100%).

**Conclusions:**

Both MT and RT were associated with high patency rates and freedom from PTS. MT may decrease the need for thrombolysis and venous stent placement. Popliteal vein thrombosis from large-bore access in MT patients requires further investigation.

**Article Highlights**

• **Type of Research:** Multicenter, retrospective, observational study
• **Key Findings:** Mechanical thrombectomy (MT) used thrombolytics less often (38% vs 95%) and required fewer stents (70% vs 98%) than rheolytic thrombectomy, with comparable 1-year secondary patency (97% in both) and similar freedom from post-thrombotic syndrome (98% vs 94%), although popliteal access site thrombosis occurred in 13% of MT patients.
• **Take Home Message:** Both thrombectomy strategies are effective for early thrombus removal; MT may offer procedural efficiencies, but necessitates careful consideration of access site thrombosis risk.

Deep vein thrombosis (DVT) remains a significant public health concern worldwide.^1^^,^^2^ The post-thrombotic syndrome (PTS) is the most frequent long-term complication of DVT and has serious implications on long-term morbidity and quality of life.^3^, ^4^, ^5^ According to recent data, approximately 5 million people in Europe are estimated to suffer from PTS.^6^ The risk of PTS after iliofemoral DVT may be as high as 50% with conservative management, with 25% of patients developing moderate to severe PTS.^6^ Among patients with inferior vena cava (IVC) thrombosis, the risk of PTS is ≤90%.^7^ Although the mainstay therapy for most proximal DVT remains anticoagulation and compression treatment,^8^, ^9^, ^10^ early thrombus removal with minimally invasive, catheter-based therapies and venous stent placement of residual venous obstruction in selected patients with symptomatic iliofemoral DVT has been shown to rapidly relieve initial symptoms and to decrease the risk of developing moderate to severe PTS.^11^, ^12^, ^13^, ^14^, ^15^

Various catheter-based treatment modalities have been developed in recent years. Standard catheter-directed thrombolysis (CDT) is effective but limited by prolonged treatment duration and coagulation monitoring in intermediate or intensive care units and may potentially increase the risk of major bleeding.^13^^,^^16^^,^^17^ In addition, CDT typically requires at least two separate procedures: initial placement of the thrombolysis catheter followed by a second look venogram with or without ballon angioplasty and stent placement. Newer percutaneous devices may have the potential to overcome some of these limitations by enabling treatment in a single session, with a potential decrease in the bleeding risk, shorter hospitalization, and the prospect of ambulatory management of DVT.^18^, ^19^, ^20^ In addition, mechanical thrombectomy (MT) and rheolytic thrombectomy (RT) can be particularly beneficial in cases where immediate restoration of blood flow is critical, such as in phlegmasia cerulea dolens, or in patients with high bleeding risk.

The ClotTriever (Inari Medical) is an MT system that consists of a thrombectomy catheter with an integrated self-expanding nitinol wire basket.^21^ The AngioJet ZelanteDVT (Boston Scientific) is an RT catheter that uses high-speed saline jets and aspiration to fragment and remove thrombus through the Venturi-Bernoulli effect with an optional thrombolytic drug delivery system (power pulse).^22^ Despite their growing use, data on the safety and efficacy of MT and RT devices remain limited. The aim of this study was to assess peri-interventional characteristics and clinical outcomes of patients with iliocaval or iliofemoral DVT treated with MT or RT.

## Methods

### Study design and patients

For the present analysis, we performed retrospective data collection on consecutive patients who underwent percutaneous thrombectomy for symptomatic iliocaval or iliofemoral DVT at two tertiary university hospitals in Zurich and Bern, Switzerland, from September 2011 to September 2024. The diagnosis was established using venous duplex ultrasound examination, with optional adjunctive axial imaging such as CT venography. The study population included patients who^1^ presented with symptomatic DVT including the iliocaval and/or iliofemoral veins,^2^ underwent percutaneous thrombus removal applying either the ClotTriever (MT group) or AngioJet ZelanteDVT (RT group),^3^ had a minimum follow-up of 3 months, and^4^ gave written informed consent for data usage. Demographic data, initial presentation (including imaging findings), venous thromboembolism risk factors, peri-interventional course, procedural data, and long-term clinical follow-up were retrospectively extracted from electronic health records. Patients were regularly followed with outpatient visits, symptom assessments, vessel patency, and complications with venous Duplex ultrasound examinations 1 day after the procedure, and then at 2 weeks, 3, 6, and 12 months, and at least once yearly thereafter. Clinical outcomes were followed to January 2025. The research project received approval from the Zurich cantonal ethics committee (Nr. 2021-00,579).

### Thrombectomy procedure

#### MT group

The ClotTriever system requires a 16F sheath placed in the popliteal vein. The catheter was inserted through the sheath over an 0.035″ guidewire, advanced beyond the thrombus, and the nitinol collection bag deployed. It was then slowly pulled back through the target thrombus toward the sheath, collecting thrombus in the collection bag. The device was cleaned and reinserted for at least four passes, each time rotated by 90° to achieve circumferential thrombectomy.

#### RT group

For AngioJet ZelanteDVT, an 8F introducer sheath was inserted into the popliteal vein, and the catheter was carefully advanced over a 0.035’’ guidewire into the thrombosed segment, while rotating the hub to achieve circumferential thrombus clearance during thrombectomy passage. Each device activation run was restricted to 30 seconds with breaks of 30 seconds in between the runs to mitigate the risk of hemolysis and bradycardic arrhythmias. If, after two passes, control venography still showed residual intraluminal thrombosis, optional recombinant tissue plasminogen activator was administered to the residual thrombus via the PowerPulse technique, with a 20-minute dwell time before a subsequent thrombectomy pass was performed The total cumulative thrombectomy time did not exceed 300 seconds.

The choice of thrombectomy device was left to the treating physician. However, the AngioJet device was mainly used before 2021 and since the introduction of the ClotTriever device in 2021, the latter has been used almost exclusively.

A staged procedure with adjunctive preinterventional CDT with a valved infusion catheter was used in patients with thrombus propagation in the popliteal vein. In patients with thrombus propagation in the IVC, either CDT or MT with optional peri-interventional implantation of an IVC filter was performed. CDT was followed by a second look venography and (pharmaco-)MT in all cases. In cases where residual stenosis exceeded 50% after thrombectomy, as determined by biplane venography, in the presence of persisting collateral flow, in the absence of flow, or in doubtful cases with intravascular ultrasound examination, adjunctive angioplasty followed by stenting using self-expandable venous stents was performed.^17^

All patients received therapeutic anticoagulation and were encouraged to wear medical compression stockings German class II (mid-tight) during the day for a minimum duration of 3 months with optional adjunctive antiplatelet therapy.

### Study outcomes

The primary clinical outcomes comprised use of periprocedural thrombolysis (ie, preprocedural or intraprocedural) and venous stents, freedom from PTS (defined as a Villalta score of <5 points^23^) and primary and secondary patency rates at 1 year. The Villalta score was routinely assessed on regular visits by a trained vascular physician. Secondary outcomes comprised sustained clinical success (defined as the absence of reintervention and the absence of PTS) and primary treatment success. In accordance with the reporting standards set forth by the Society of Interventional Radiology,^24^ primary treatment success was defined as successful restoration of antegrade in-line flow in the treated vein with elimination of any underlying obstructive lesion at the end of the final endovascular procedure. The primary patency rate was defined as the percentage of patients with primary treatment success and without the occurrence of either thrombosis of the treated segment or a reintervention to maintain patency of the treated segment. The primary-assisted patency rate was defined as the percentage of patients with primary treatment success and without the occurrence of thrombosis of the treated segment, irrespective of any interval therapy to restore or maintain flow within the treated segment. The secondary patency rate was defined as the percentage of patients with primary treatment success and without the occurrence of permanent loss of flow in the treated segment, irrespective of any interval therapies.

Patency of the treated segment was evaluated using duplex ultrasound examination, using the following criteria. (1) Vessel occlusion was defined as the complete absence of both color flow and pulsed-wave Doppler signal within the treated stent segment, indicating total loss of luminal patency. (2) Vein stenosis of >50% were diagnosed according to the previously published stenosis criteria of iliocaval venous stents,^25^ and comprised indirect criteria (any Doppler flow pattern other than spontaneously modulated by respiration or peak flow velocity of ≤10 cm/s at the stent inlet) and direct criteria (ratio of in-stent to stent-inlet peak velocity of ≥3.5 or a peak flow velocity in stent of >44 cm/s).^25^ Primary safety outcomes were device-related adverse events and major bleeding. We used modified definition criteria of the reporting standards of the International Society on Thrombosis and Haemostasis to assess major bleeding complications, which include fatal bleeding, symptomatic bleeding in a critical organ, or bleeding that necessitates the transfusion of ≥2 U whole blood or red cells.^26^ A device-related adverse event was defined as any detrimental or unintended consequence arising from the use, malfunction, or misuse of a medical device.

### Statistical analysis

Continuous variables are expressed as means ± standard deviations for normally distributed data or as medians with interquartile ranges (first quartile [Q1], third quartile [Q3]) for non-normally distributed data. Categorical variables are expressed as absolute numbers and percentages, and categorical outcomes as percentage with 95% confidence intervals (CIs). Comparisons between the two groups were performed using an unpaired *t* test or the Wilcoxon-Mann-Whitney *U* test for continuous variables, and the χ^2^ test or Fisher's exact test for categorical variables. Patency rates were calculated using the Kaplan-Meier survival analysis and compared by use of univariate Cox regression analysis. A *P* value of <.05 was considered statistically significant. Statistical analyses were conducted using IBM SPSS version 29.0 (IBM Corp).

## Results

### Study population

A total of 122 patients (mean age, 48 years; 57% women) were included in the study: 40 (33%) received MT and 82 (67%) RT. Demographics, comorbidities, and venous thromboembolism risk factors are shown in Table I. The incidence of bilateral iliac DVT (33% vs 1%) and IVC thrombosis (43% vs 12%) was higher in the MT group than in the RT group. Among patients treated with MT, acute on chronic DVT secondary to IVC atresia occurred in 20%, whereas no cases of IVC atresia were identified in the RT group.

**Table I:** Baseline characteristics

	Total (n = 122)	MT (n = 40)	RT (n = 82)
Demographics			
Age, years	47.7 ± 20.3	46.1 ± 20.1	48.5 ± 20.0
Female sex	69 (56.6)	19 (47.5)	50 (61.0)
Comorbidities and risk factors			
Immobilization (last 3 months)	27 (22.1)	6 (15.0)	21 (25.6)
Estrogen therapy/hormonal contraception	26 (21.3)	3 (7.5)	23 (28.0)
Hospitalization (last 6 months)	20 (16.4)	4 (10.0)	16 (19.5)
Major surgery (last 3 months)	10 (8.2)	2 (5.0)	8 (9.7)
Trauma	6 (4.9)	0 (0.0)	6 (7.3)
Varicose veins	15 (12.3)	3 (7.5)	12 (14.6)
Active cancer	7 (5.7)	3 (7.5)	4 (4.9)
Previous VTE	19 (15.6)	3 (7.5)	16 (19.5)
VTE details			
Left sided	86 (70.5)	15 (37.5)	71 (86.6)
Iliac vein compression	57 (46.7)	12 (30.0)	45 (54.9)
Isolated iliac DVT	16 (13.1)	4 (10)	12 (14.6)
Symptomatic concomitant PE	22 (18)	7 (17.5)	15 (18.3)
Duration of symptoms			
Acute (≤14 days)	117 (95.9)	35 (87.5)	82 (100.0)
Subacute/chronic (>14 days)	5 (4.1)	5 (12.5)	0 (0.0)
Thrombosed vein segments			
IVC	27 (22.1)	17 (42.5)	10 (12.2)
CIV	105 (86.1)	37 (92.5)	68 (82.9)
EIV	120 (98.4)	40 (100.0)	80 (97.6)
CFV	107 (87.7)	36 (90.0)	71 (86.6)
FV	67 (54.9)	22 (55.0)	45 (54.9)
DFV	44 (36.1)	17 (42.5)	27 (32.9)
PV	28 (23.0)	10 (25.0)	18 (22.0)
Below-the-knee (calf) veins	9 (7.4)	3 (7.5)	6 (7.3)

*CFV,* Common femoral vein; *CIV,* common iliac vein; *DFV,* deep femoral vein; *DVT,* deep vein thrombosis; *EIV,* external iliac vein; *FV,* femoral vein; *IVC,* inferior vena cava; *MT,* mechanical thrombectomy; *PE,* pulmonary embolism; *PV,* popliteal vein; *RT,* rheolytic thrombectomy; *VTE,* venous thromboembolism.

Values are mean ± standard deviation or number (%).

### Treatment details

Primary treatment success was achieved in all patients. Overall, single session thrombectomy (without prior CDT use) was performed in 67 patients (55%): 27 (68%) in the MT group and 40 (49%) in the RT group (*P* = .17). Among the 55 patients (45%) who underwent CDT followed by staged bailout thrombectomy, additional intraprocedural thrombolytic therapy was administered in 0% of MT cases compared with 50% of RT cases (*P* < .01). Table II displays detailed information on thrombolytic use. Protected thrombectomy with the insertion of an IVC filter was performed in five patients (13%), all in the MT group. In four cases, the IVC filter was retrieved at the end of the procedure; in one case, retrieval was delayed until after 5 days of anticoagulation owing to thrombus detected within the filter during the intervention. Overall, provisional venous stent placement after ballon angioplasty was performed in 108 patients (89%): 28 (70%) in the MT group and 80 (98%) in the RT group (*P* < .01). After thrombectomy, 57 patients (47%) had an underlaying iliac vein compression: 12 (30%) in the MT and 45 (55%) in the RT group (*P* = .017). In patients with iliac vein thrombosis extending down to the femoral veins, stent extension across the inguinal ligament was necessary in 31 patients (25%): 6 (17%) in the MT group and 25 (36%) in the RT group owing to incomplete thrombus clearance (*P* = .044). Procedural details are provided in Table III.

**Table II:** Thrombolysis use

	Total (n = 122)	MT (n = 40)	RT (n = 82)	*P* value
Thrombolysis (r-tPA) use	93 (76.2)	15 (37.5)	78 (95.1)	<.0001
CDT before thrombectomy	55 (45.1)	13 (32.5)	42 (51.2)	.056
r-tPA via CDT per intervention, mg	23.1 ± 7.4	25.5 ± 9.4	22.4 ± 6.6	-
Use of r-tPA during thrombectomy session	61 (50)	2 (5)	59 (72)	<.0001
Total r-tPA per intervention, mg	19.9 ± 10.5	23.4 ± 10.3	19.2 ± 10.5	-

*CDT,* Catheter-directed thrombolysis; *MT,* mechanical thrombectomy; *RT,* rheolytic thrombectomy; *r-tPA,* recombinant tissue plasminogen activator.

Values are number (%) or mean ± standard deviation.

**Table III:** Procedural data

	Total (n = 122)	MT (n = 40)	RT (n = 82)	*P* value
Implanted stents, n	1.3 ± 1.0	1.3 ± 1.4	1.3 ± 0.6	-
Stented vein segments				
IVC	13 (10.7)	12 (30.0)	1 (1.2)	<.0001
CIV	92 (75.4)	25 (62.5)	67 (83.8)	.026
EIV	92 (75.4)	23 (57.5)	69 (84.1)	.003
CFV	35 (28.7)	6 (15.0)	29 (35.4)	.02
FV or DFV	3 (2.5)	0 (0.0)	3 (3.7)	.55

*CFV,* Common femoral vein; *CIV,* common iliac vein; *DFV,* deep femoral vein; *EIV,* external iliac vein; *FV,* femoral vein; *IVC,* inferior vena cava; *MT,* mechanical thrombectomy; *RT,* rheolytic thrombectomy.

Values are mean ± standard deviation or number (%).

Peri-interventional major bleeding complications occurred in one patient in the RT group with intra-abdominal bleeding. The patient underwent CDT before thrombectomy with a cumulative dose of 40 mg alteplase. Access site thrombosis of the popliteal vein occurred in five patients (13%) in the MT group, whereas no such cases were observed in the RT group (*P* < .01). Early stent occlusion within 7 days occurred in two patients (2%), both in the RT group.

### Stent patency rates and clinical outcomes

Overall, the median follow-up was 25 months (interquartile range [IQR], 11-52 months): it was 13 months (IQR, 4-27 months) in the MT group and 39 months (IQR, 17-61 months) in the RT group (*P* < .01), reflecting the different time window of device availability and clinical use.

Overall, the primary patency rate at 12 months was 85% (95% CI, 79%-92%): it was 80% (95% CI, 67%-95%) in the MT group and 88% (95% CI, 81%-96%) in the RT group. The assisted primary patency rate at 12 months was 91% (95% CI, 86%-97%): it was 92% (95% CI, 84%-100%) in the MT group and 90% (95% CI, 85%-96%) in the RT group. The secondary patency rate at 12 months was 97% (95% CI, 94%-100%): it was 97% (95% CI, 93%-100%) in the MT group and 97% (95% CI, 94%-100%) in the RT group (Fig).

**Fig:**
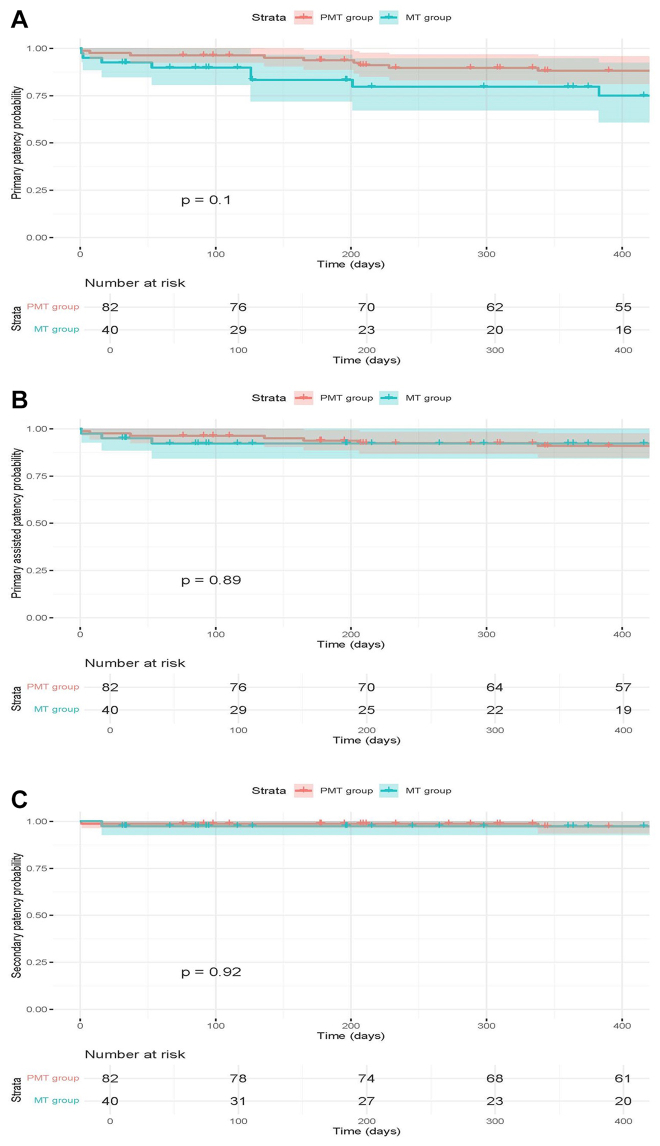
Kaplan-Meier analysis of primary **(A)**, primary-assisted **(B)**, and secondary **(C)** patency rates.

Overall, sustained clinical success at last follow-up was achieved in 100 patients (82%): 32 (80%) in the MT and 68 (83%) in the RT group. Overall, six patients (5%) developed the PTS at latest follow-up: one (3%) in the MT group and five (6%) in the RT group (four missing data). No patient developed severe PTS or leg ulcers. Three patients (8%) in the MT group who did not initially receive a stent after primary thrombectomy required subsequent stent placement owing to persistent symptomatic iliac vein stenosis. Adverse events and clinical outcomes are detailed in Table IV.

**Table IV:** Adverse events and clinical outcomes

	Total (n = 122)	MT (n = 40)	RT (n = 82)
Patients with adverse events			
Iliacofemoral stent/vein occlusion	12 (9.8) [95% CI, 1.4% to 9.3%]	3 (7.5) [95% CI, 1.9% to 19.8%]	9 (11.0) [95% CI, 1% to 10.9%]
Iliacofemoral stent/vein occlusion (with ≥1 reintervention)	9 (7.4) [95% CI, 3.3% to 11.5%]	2 (5.0) [95% CI, 0.7% to 12.0%]	7 (8.5) [95% CI, 3.4% to 13.6%]
Permanent stent/vein occlusion	3 (2.5) [95% CI, 0.6% to 6.1%]	1 (2.5) [95% CI, 0.0% to 7.5%]	2 (2.4) [95% CI, 0.0% to 5.8%]
Iliacofemoral stent stenosis (with reintervention)	3 (2.5) [95% CI, 0.6% to 6.1%]	1 (2.5) [95% CI, 0.0% to 7.5%]	2 (2.4) [95% CI, 0.0% to 5.8%]
Patients with (recurrent) VTE			
DVT outside stented segment	6 (4.9) [95% CI, 1.5% to 8.3%]	5 (12.5) [95% CI, 4.1% to 21.2%]	1 (1.2) [95% CI, −0.7% to 3.1%]
Patients with bleeding			
Major bleeding	1 (0.8) [95% CI, 0.0% to 2.3%]	0 (0.0) [95% CI, 0.0% to 8.1%]	1 (1.2) [95% CI, 0.0% to 3.4%]
Minor bleeding	8 (6.6) [95% CI, 3.4% to 9.8%]	3 (7.5) [95% CI, 1.7% to 13.3%]	5 (6.1) [95% CI, 2.0% to 10.1%]
Clinical outcomes^a^			
Median Villalta score at last follow-up (IQR)	0 (2)	1 (2)	1 (2)
No PTS (<5 points)	116 (95.1) [95% CI, 89.8% to 100%]	39 (97.5) [95% CI, 92.6% to 100%]	77 (93.9) [95% CI, 87.4% to 100%]
Mild PTS (5-9 points)	4 (3.3) [95% CI, 0.2% to 6.4%]	1 (2.5) [95% CI, 0.0% to 7.3%]	3 (3.6) [95% CI, 0.0% to 8.4%]
Moderate PTS (10-14 points)	2 (1.6) [95% CI, 0.0 to 3.9%]	0 [95% CI, 0.0,7.5%]	2 (2.4) [95% CI to 0.0, 5.7%]
Severe PTS (≥15 points or venous ulcer)	0 (0)	0 (0)	0 (0)
Missing data	4 (3.2)	2 (5.2)	2 (2.5)

*CI,* Confidence interval; *DVT,* deep vein thrombosis; *IQR,* interquartile range; *MT,* mechanical thrombectomy; *PTS,* post-thrombotic syndrome; *RT,* rheolytic thrombectomy; *VTE,* venous thromboembolism.

Values are number (%) unless otherwise indicated.

a Incidence of postthrombotic syndrome based on Villalta score at the last follow-up.

## Discussion

This study provides a comparison of mid-=term clinical outcomes for two endovascular thrombectomy approaches in patients with iliocaval and iliofemoral DVT: MT with the ClotTriever and RT with the AngioJet ZelanteDVT. In this study, both MT and RT showed favorable clinical outcomes: only about 5% of patients developed (mostly mild) PTS. Similar results were reported in the CLOUT (ClotTriever Outcomes) registry. In this study, 19% of patients with acute DVT developed PTS at the 1-year follow-up after MT with the ClotTriever.^21^ Similar rates (4%-6%) were described in retrospective studies on RT with the AngioJet ZelanteDVT.^22^^,^^27^ The low PTS rates may be explained by the high primary and secondary patency rates (>97%). Indeed, 6-month patency was a key predictor of the PTS in the randomized CaVenT (Catheter-directed Venous Thrombolysis) study.^21^^,^^28^ A subgroup analysis of patients with iliofemoral DVT included in ATTRACT (Acute Venous Thrombosis: Thrombus Removal with Adjunctive Catheter-Directed Thrombolysis) showed higher rates of PTS in the interventional group at 2 years of follow-up at 33%; however, the study had no duplex ultrasound follow-up.^29^

As compared with anticoagulation therapy alone, the use of thrombolysis increases the risk of major bleeding complications among DVT patients.^29^, ^30^, ^31^ In the present study, periprocedural thrombolysis was used less frequently in patients treated with MT (38% vs 95%; *P* < .01), although the MT group showed a greater thrombus burden, including thrombus extending to the IVC. Furthermore, 67% of MT patients were managed in a single session without CDT as opposed to 50% in the RT group. Our data are in line with Abramowitz et al,^32^ who showed higher rates of single-session treatment for patients treated with MT. In contrast, single-session treatment was more often used in our RT group (50%) than in the multicenter PEARL (Peripheral Use of AngioJet Rheolytic Thrombectomy with a Variety of Catheter Lengths) registry (39%); however, overall thrombolysis use was very similar (95% in our study vs 96% in the PEARL registry).^33^ In our opinion, CDT plus optional stent placement in a second session remains a good treatment option for free-floating IVC thrombus or for patients with thrombosed inflow veins, including the popliteal vein and below-the-knee veins. The ideal indication for single-session MT or RT remains descending DVT with proximal compression or stenosis of the iliac vein or the IVC and good inflow with a patent popliteal vein.

The overall stent angioplasty rate was significantly lower after MT than after RT (70% vs 98%; *P* < .01). The ClotTriever can efficiently remove subacute and more organized thrombi.^34^, ^35^, ^36^ Of note, thrombus age may be difficult to predict based on the duration of symptoms.^37^ The CLOUT registry reported an even lower (44.3%) stent placement rate after MT, likely owing to a lower thrombus burden. In comparison with our MT patients, CLOUT patients more often had isolated femoropopliteal DVT (18% vs 0%) and less often had iliofemoral DVT (82% vs 100%) and bilateral DVT (4% vs 33%). None of the CLOUT patients had IVC thrombosis (0% vs 43%).^21^^,^^38^ However, the stent placement rate in the subgroup of our patients with descending DVT and compression of the left common iliac vein was high and similar in both groups (100% for RT vs 91.7% for MT). As compared with RT, stent extension to the common femoral vein was less often performed in MT patients (17% vs 36% with RT; *P* = .044) for the same reason of more effective removal of organized subacute thrombus with the ClotTiever.

Both thrombectomy systems were safe with a low rate of major and minor bleedings in both groups. However, five MT patients (12.5%) vs no RT patient suffered access site thrombosis of the popliteal vein, diagnosed by systematic duplex ultrasound examination on the day after intervention, of which 40% extended up to the femoral vein. No puncture site complications were registered in the RT group. The MT device requires a larger (16F) sheath as compared with the RT device (8F), which is the most likely reason for this observation. Nevertheless, none of these patients presented with PTS at the last follow-up. Future studies should include early duplex ultrasound evaluation of the popliteal vein to rule out venous access site thrombosis.

This study has several limitations. First, the retrospective nature may introduce selection bias limiting generalizability. Second, owing to the nonrandomized design, differences in patient characteristics, thrombus burden, device availability, and evolving stent placement practices may have influenced treatment selection; therefore, results between groups should be interpreted with caution. Third, follow-up was limited to a median duration of 25 months and was different in the two groups owing to more recent market introduction of the MT device, which may have resulted in underdetection of late PTS and stent-related complications in this group. Nevertheless, most patients had >1 year of clinical and duplex follow-up. Prospective, multicenter studies are warranted to validate our findings and investigate patient-centered outcomes such as quality of life and functional recovery.

## Conclusions

In this real-world cohort, both MT and RT were associated with favorable clinical outcomes and patency rates in patients with symptomatic iliocaval and iliofemoral DVT. In clinical practice, MT was commonly applied as a single-session approach, was frequently performed with limited use of thrombolysis, and often required fewer stent extensions to address residual post-thrombotic inflow vein changes. Future studies should investigate the risk of access site thrombosis of the popliteal vein for MT devices requiring large venous access.

## Author contributions

Conception and design: GF, SB, NK, RE

Analysis and interpretation: GF, TS, DP, SB, RE

Data collection: GF, SC, RF

Writing the article: GF, SB, NK, RE

Critical revision of the article: GF, SC, RF, TS, DP, SB, NK, RE

Final approval of the article: GF, SC, RF, TS, DP, SB, NK, RE

Statistical analysis: Not applicable

Obtained funding: Not applicable

Overall responsibility: GF

## Funding

None.

## Disclosures

N.K. received grant support from the Swiss National Science Foundation, Concept Medical, Bayer, Becton Dickenson, Boston Scientific, and Medtronic. He also received consultancy or lecturer fees from Boston Scientific and Becton Dickenson.
